# Supplementary material for: Narrow-spectrum resource-utilizing bacteria drive the stability of synthetic communities through enhancing metabolic interactions
Source: Nat Commun. 2025 Jul 2;16:6088. doi: 10.1038/s41467-025-61432-7 (PMC12222865; doi:10.1038/s41467-025-61432-7)
Supplement: Supplementary file 1 — Supplementary Information [file 41467_2025_61432_MOESM1_ESM.pdf]

Narrow-spectrum resource-utilizing bacteria drive the stability of synthetic communities through enhancing metabolic interactions

Wang *et al.*

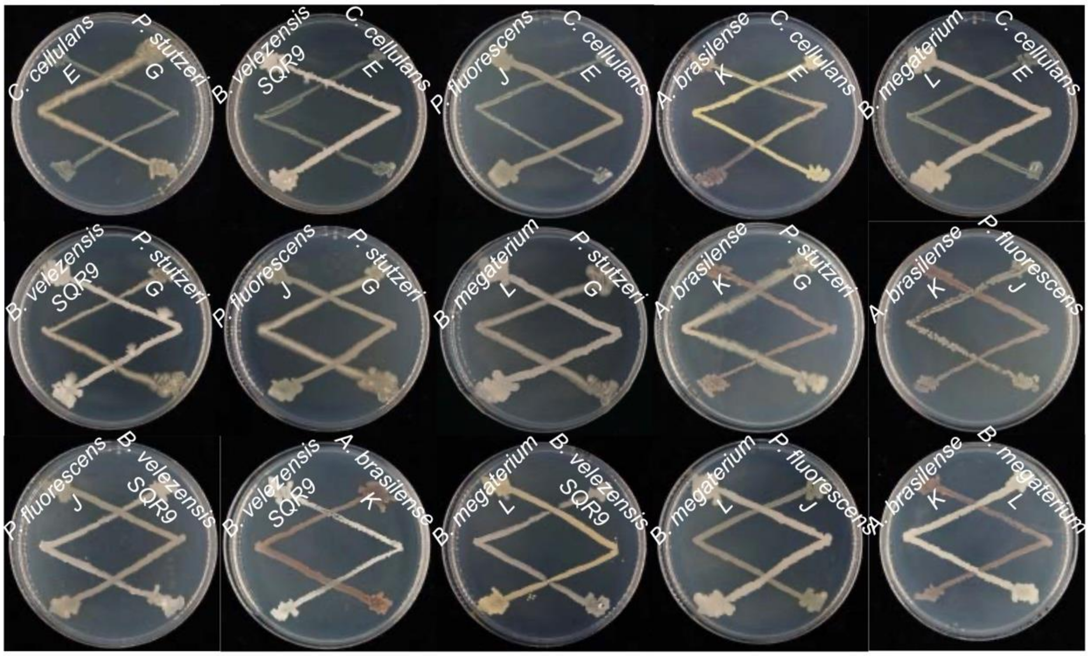

Supplementary Fig. 1. The contact antagonism relationships between strains. Strain pairs of six plant-beneficial bacteria were cross-streaked on RE medium plates and incubated at 30 ℃ for 48 h. The merging of the bacterial colonies indicates compatibility among the bacterial strains.

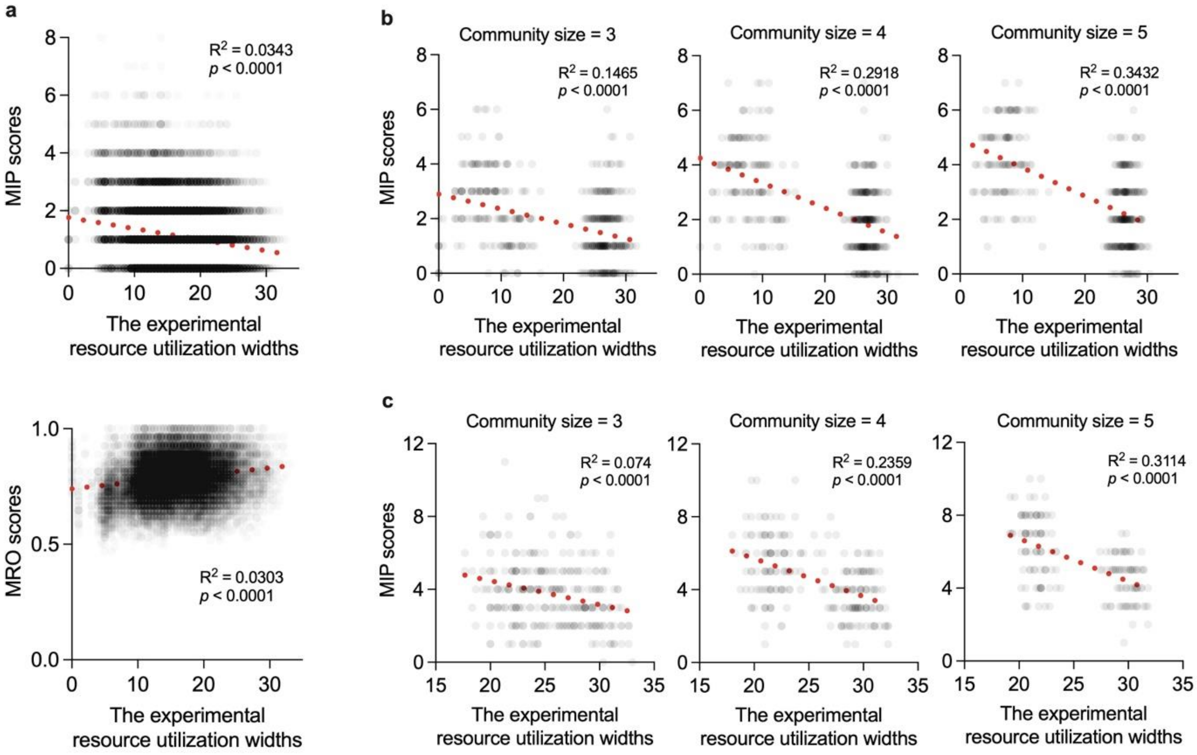

Supplementary Fig. 2. Relationships between resource utilization width and community MIP/MRO in plant-associated bacteria. (**a**) Correlation between average experimental resource utilization widths and community metabolic interaction potential (MIP) or metabolic resource overlap (MRO) scores across all 24,976 pairwise interaction simulations of 224 phyllosphere strains. Correlations between average experimental utilization widths and MIP scores in 3-, 4-, and 5-member communities constructed from either 224 phyllosphere strains (**b**) or 25 laboratory-stored rhizosphere strains (**c**). The line represents a linear regression fit. Source data are provided as a Source Data file.

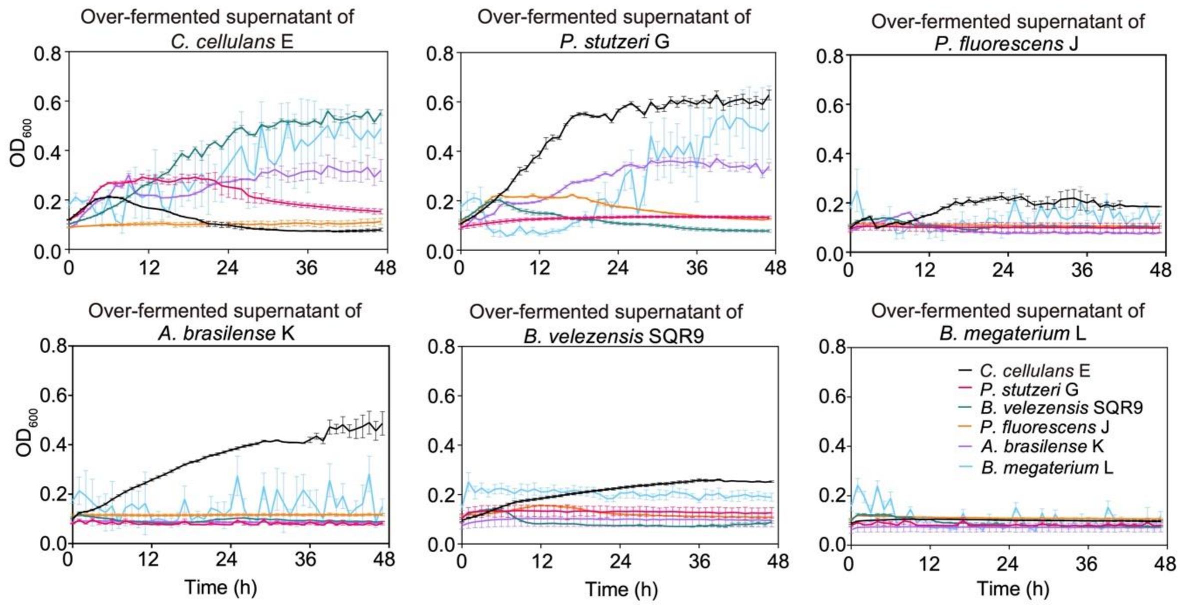

Supplementary Fig. 3. Monoculture growth curves on post-fermentation supernatants of other strains. Post-fermentation supernatants of each strain cultured in RE medium were used for the continued incubation of the other five strains. Error bars represent mean ± SD of biological replicates (n = 5). Source data are provided as a Source Data file.

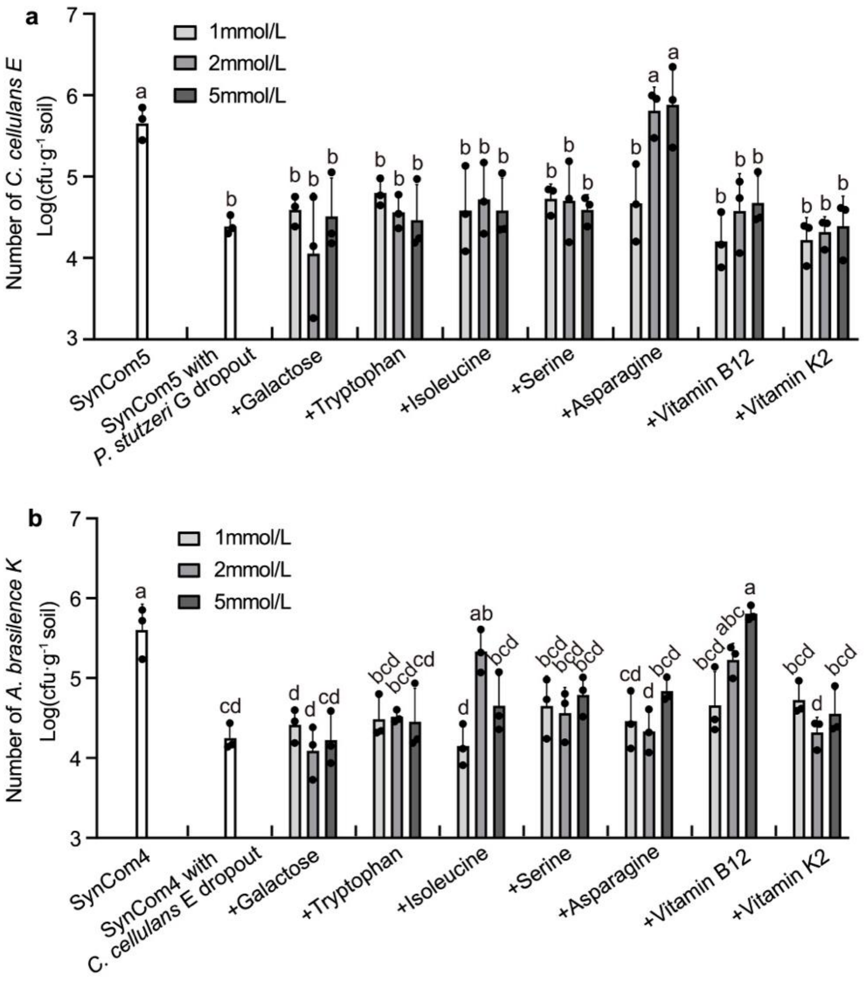

Supplementary Fig. 4. **Metabolite supplementation validation for SynCom4 and SynCom5.** Predicted key metabolites, including galactose, tryptophan, isoleucine, serine, asparagine, vitamin B12, and vitamin K2, were supplemented in SynCom5 lacking *P. stutzeri G* (SynCom5-*P. stutzeri* G) and SynCom4 lacking *C. cellulans* E (SynCom4-*C. cellulans* E). The impact of metabolite supplementation on the rhizosphere abundance of *C. cellulans* E in SynCom5 (**a**) and *A. brasilense* K in SynCom4 (**b**), both of which reduced due to strain dropout, was assessed. Different letters represented a significantly difference ($p < 0.05$, one-way ANOVA, two-sided), and error bars represent mean ± SD of biological replicates (n = 3). Source data are provided as a Source Data file.

Supplementary Table 1. Strain-specific primers used in this study.

Strain		Primer sequences (5'-3')
C. cellulans E	forward	ACTCAACGACTCCATCTACAA
	reverse	GTTGATGTAGAGCCAGATGTC
A. brasilense K	forward	TTCTCCAGCTACATGATTCAG
	reverse	GTAATAGACGCCTTCCTTCTC
P. stutzeri G	forward	CAGAACACCTACAAAACACTCG
	reverse	GTTCTTCAGCCGGTACTTGT
B. velezensis SQR9	forward	GGAATACTGGCAGGAATGG
	reverse	GTCCGTATGATTGAGAGGTT
B. megaterium L	forward	GAACTAAGGATAGGGGAGCTTAC
	reverse	GACTAGACCGTCTTGCTTAACTC
P. fluorescens J	forward	GTTGTTCAATGAGGAAGTGT
	reverse	AGCAAGGTGTAGTTGTCAA
