# Supplementary material for: Narrow-spectrum resource-utilizing bacteria drive the stability of synthetic communities through enhancing metabolic interactions
Source: Nat Commun. 2025 Jul 2;16:6088. doi: 10.1038/s41467-025-61432-7 (PMC12222865; doi:10.1038/s41467-025-61432-7)
Supplement: Supplementary file 3 — Description of Additional Supplementary Files [file 41467_2025_61432_MOESM3_ESM.pdf]

Description of Additional Supplementary Files

File Name: Supplementary Data 1
Description: Carbon source utilization profiles of 25 rhizosphere bacteria.

File Name: Supplementary Data 2
Description: Untargeted metabolomic analysis of six individual strains and their respective synthetic communities.
